# Issues Associated With the Use of Semantic Web Technology in Knowledge Acquisition for Clinical Decision Support Systems: Systematic Review of the Literature

**DOI:** 10.2196/medinform.6169

**Published:** 2017-07-05

**Authors:** Seyedjamal Zolhavarieh, David Parry, Quan Bai

**Affiliations:** ^1^ Department of Computer Science Auckland University of Technology Auckland New Zealand

**Keywords:** semantic web technology, clinical decision support system, systematic review, medical informatics, knowledge, Internet

## Abstract

**Background:**

Knowledge-based clinical decision support system (KB-CDSS) can be used to help practitioners make diagnostic decisions. KB-CDSS may use clinical knowledge obtained from a wide variety of sources to make decisions. However, knowledge acquisition is one of the well-known bottlenecks in KB-CDSSs, partly because of the enormous growth in health-related knowledge available and the difficulty in assessing the quality of this knowledge as well as identifying the “best” knowledge to use. This bottleneck not only means that lower-quality knowledge is being used, but also that KB-CDSSs are difficult to develop for areas where expert knowledge may be limited or unavailable. Recent methods have been developed by utilizing Semantic Web (SW) technologies in order to automatically discover relevant knowledge from knowledge sources.

**Objective:**

The two main objectives of this study were to (1) identify and categorize knowledge acquisition issues that have been addressed through using SW technologies and (2) highlight the role of SW for acquiring knowledge used in the KB-CDSS.

**Methods:**

We conducted a systematic review of the recent work related to knowledge acquisition MeM for clinical decision support systems published in scientific journals. In this regard, we used the keyword search technique to extract relevant papers.

**Results:**

The retrieved papers were categorized based on two main issues: (1) format and data heterogeneity and (2) lack of semantic analysis. Most existing approaches will be discussed under these categories. A total of 27 papers were reviewed in this study.

**Conclusions:**

The potential for using SW technology in KB-CDSS has only been considered to a minor extent so far despite its promise. This review identifies some questions and issues regarding use of SW technology for extracting relevant knowledge for a KB-CDSS.

## Introduction

Decision-making is an essential activity for clinicians. In this paper, we are primarily concerned with knowledge-based diagnostic decisions. Other decisions include image interpretation, drug discovery, and others. Such decisions are clearly critical. Clinical decision-making is a daily process for all practitioners making decisions about patient care. The quality of decisions depends on how much experience experts have and how much accurate knowledge is available. Clinical diagnostic decision-making is a complex activity and requires clinicians to have access to relevant, up-to-date, and accurate knowledge sources to support appropriate patient care. A knowledge-based clinical decision support system (KB-CDSS) uses machine-stored knowledge to assist clinicians. Other CDSSs may learn from large amounts of data via machine learning techniques or act as a case-based reasoning system [1].

Recently, Informatics researchers have proposed several computerized methods to find relevant and accurate knowledge to assist in diagnosis. KB-CDSS requires knowledge to be available, rather than generating its own knowledge through machine learning. Knowledge-based approaches may be more effective in cases where little data is available, or there is a need for explanatory capacity. Early decision support systems such as MYCIN [2] used knowledge-based approaches, albeit from knowledge collected by experts. However, there are limitations in their use in terms of the need to fit together with the use of clinical experience. KB-CDSSs may be most useful where the clinician does not have recent experience of a particular problem or may not feel that their knowledge is up to date.

The core of each KB-CDSS contains three components including a central knowledge base (KB), an inference or reasoning engine, and a user or communication interface [3]. The KB-CDSS receives patient data and inputs and provides a diagnosis as an output. In this regard, the KB plays a vital role in this scenario for collecting, classifying, and sharing the knowledge [4].

The KB-CDSS works by extracting knowledge from various knowledge sources. However, knowledge acquisition is one of the well-known bottlenecks for any kind of KB-CDSS. Providing an intelligent mechanism for communicating between KB-CDSSs and knowledge resources is a major concern of today’s researchers since inappropriate or low-quality knowledge may not give appropriate outputs. More precisely, the KB-CDSS cannot be effective if it uses limited or outdated knowledge in response to a given query about a particular disease or set of symptoms [5].

Semantic Web (SW) technology is an effort to make knowledge on the Web both human-understandable and machine-readable [6]. In the context of KB-CDSSs, there is well-known biomedical research that has used SW technologies [7-9] and semantic mechanisms [10,11] to improve the process of knowledge acquisition in KB-CDSSs [12-15]. However, it is still unclear how SW technologies can be efficiently used to support KB-CDSS knowledge acquisition. For example, the quality of extracted knowledge has not been evaluated yet, even in the SW-based KB-CDSSs.

Recently, there has been exponential growth in the amount of published medical knowledge. For example, PubMed has grown by around 4% a year and contains more than 20 million articles [16]. Available knowledge resources are very diverse in terms of formats, structure, and vocabulary. Since 2005, researchers have been developing SW-based KB-CDSSs to effectively extract knowledge from such heterogeneous environments [15,17-20].

This paper is a systematic review that aims to identify and describe the knowledge acquisition issues related to SW technologies for KB-CDSSs. It attempts to classify the issues discovered and offer suggestions for open questions in this field.

## Methods

### Search Criteria and Selection

In this study, a systematic review framework was applied to search, extract, and assess articles. We used a keyword search strategy to find relevant articles that contain “Semantic Web Technology” and “Clinical Decision Support System” (see Table 1). SW technologies started to be used to support KB-CDSSs [21] after 2005. In order to extract related articles, we queried PubMed, Web of Science, Journal of Biomedical Informatics, Knowledge and Information Systems, Journal of Medical Systems, Artificial Intelligence in Medicine, Current Bioinformatics, Journal of Convergence Information Technology (JCIT), eHealth Networking Application and Services, and Health Science.

**Table 1:** Clinical variables among responders by type of diabetes.

Search lines	Search terms	Filtered by
Line 1	“Semantic technology” OR “Semantic Web technology” OR “Semantic Web” OR “Semantic Web techniques” OR “Semantic-based” OR “Semantic-Web-based.”	Title or abstract
2. AND	“Clinical Decision Support” OR “Clinical Decision making” OR “Medical Decision Support” OR “Medical Decision making” OR “Clinical Decision Support System” OR “Medical Decision Support System” OR “CDS” OR “CDSS.”	Title or abstract
3. AND	“Architecture” OR “Framework” OR “System” OR “Model.”	Title or abstract
4. AND	“Health” OR “disease” OR “case study” OR “public health.”	Title or abstract
5. AND	“Diagnosis” OR “treatment” OR “prediction” OR “reasoning.”	Title or abstract

## Results

### Recovered Documents

**Figure 1:**
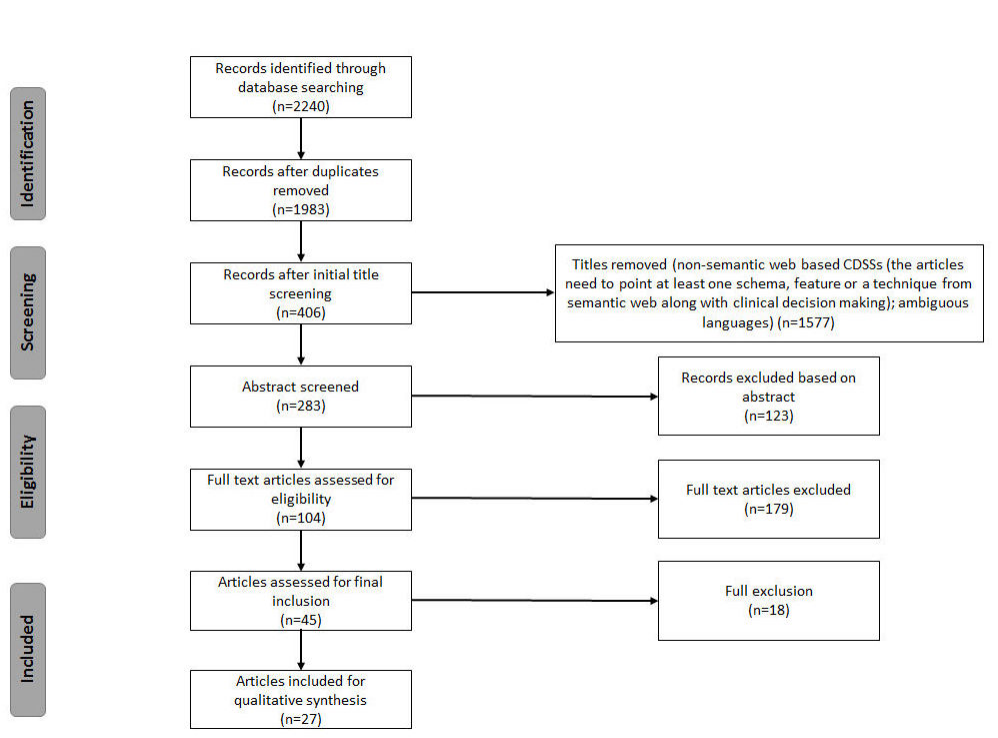
Flow diagram showing the exclusion of articles at various stages of the research.

Details about the inclusion and exclusion of articles are provided in Figure 1. These queries returned 2240 articles. This is a result of querying “Semantic Web Technology” and “Clinical Decision Support System” together. We then checked the titles and abstracts to refine the set. By checking the title or abstract, we reduced the number of articles to 283. In the general selection phase, we considered those papers that pointed to the concepts of SW technologies and CDSSs together. In this regard, there is a large number of articles that discuss CDSSs. However, few of these articles review the importance and benefit of SW technologies in the area of KB-CDSSs. We only considered papers that strongly focused on improving knowledge acquisition issues in the context of KB-CDSSs through applying SW technologies. We also excluded articles if they were not in the English language. Of the remaining 283 articles, 27 met the inclusion criteria.

### Overview

To achieve a better understanding of knowledge acquisition issues that have been addressed using SW technologies, we categorized articles based on two main issues: (1) format and data heterogeneity and (2) lack of semantic analysis. In Textboxes 1 and 2, we describe the issues along with the number of papers that focus on the issues (n/N).

**Format and data heterogeneity issues (18/27).**

Format and data heterogeneity were divided into the following subcategories:

- Format heterogeneity: This issue comes from different ways of representing and storing the same data. Due to the inconsistency among data models, connecting different biomedical knowledge sources is not an easy task.
- Data heterogeneity: This issue refers to the redundant results for the same entry such as having multiple entries for the same data.
- Lack of data integration: This issue is related to the lack of having a unified model for combining data residing in different sources. In this regard, clinical health care systems need a unified model to share and reuse knowledge among each other.

**Lack of semantic analysis issues (9/27).**

Lack of Semantic Analysis was divided into two subcategories:

- Weak semantic infrastructure: Lack of a semantic infrastructure, effectively shared understanding of meaning, reduces the value of results of queries from health care knowledge sources. Resource Description Framework (RDF) is a unified data model proposed by the Semantic Web community that is useful for remedying such an issue.
- Lack of semantic definitions: Without sufficient semantic definitions, knowledge-based clinical decision support systems are not able to interpret the meaning of extracted knowledge. Such knowledge is usually encoded in the ontology (ie, schema-level) which is the backbone of Semantic Web.

**Figure 2:**
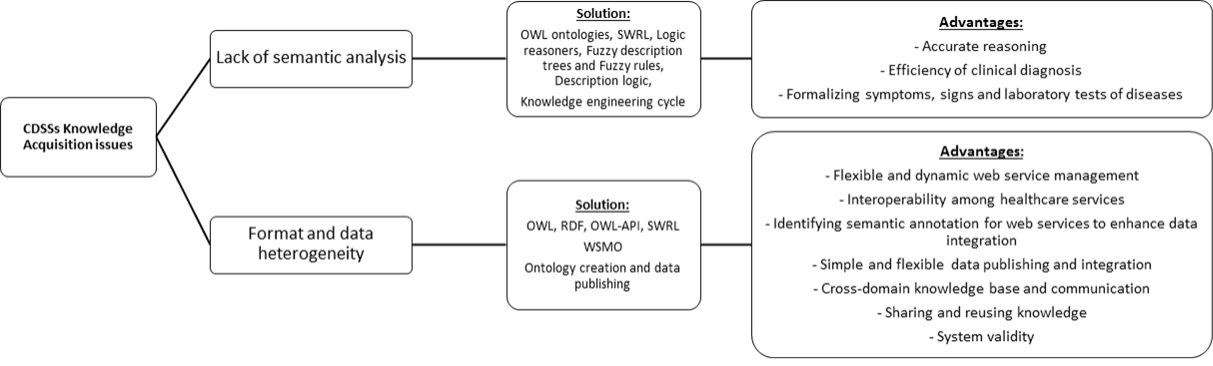
Knowledge acquisition issues of clinical decision support systems (CDSSs) improved by Semantic Web (SW) technologies.

This review discovered that the most important issue is related to format and data heterogeneity. Figure 2 depicts how using SW technologies helps to remedy the issues. SW technology is an efficient way to improve knowledge acquisition for several reasons such as providing an intelligent query processing mechanism rather than keyword-based answering process, providing an easy inference process, organizing the knowledge in conceptual domains, supporting consistency, facilitating knowledge extraction, supporting data integration as well as semantic interoperability, providing knowledge retrieval, and knowledge representation. In the following, we review the recent related work, which deal with SW technologies for remedying the knowledge acquisition of KB-CDSSs for the purpose of diagnosis.

### Format and Data Heterogeneity

It is important to mention that the reviewed papers proposed two different types of frameworks to overcome the issues. These frameworks, which have been developed by utilizing SW technologies, are ontological-based structures and SW services. In this regard, SW technologies have been used to boost the process of knowledge acquisition in KB-CDSSs.

#### Ontological-Based Structures

By using emerging SW technologies such as Resource Description Framework (RDF), Web Ontology Language (OWL), and Semantic Web Rule Language (SWRL), researchers have started to utilize SW technologies to empower and facilitate the process of knowledge sharing among KB-CDSSs. An ontology is potentially very useful in SW as it identifies the relationships between concepts in a domain. One of the most popular approaches for reducing the problem of data and format heterogeneity of KB-CDSSs is therefore to use an ontological-based structure.

[22] addressed antimicrobial health problems and inappropriate antibiotic prescribing in the health care domain. In this study, an application-independent KB-CDSS model has been developed by using formal ontological methods. The method used some SW standardizations such as OWL and SWRL to evaluate the results through intrinsic and extrinsic evaluation studies. However, this study suffers from the lack of having an accurate evaluation mechanism. The results of the study were mostly gathered in a laboratory setting rather than a clinical setting.

[23] proposed an ontological-mediated decision support system for breast cancer by utilizing SW technologies to use data for the decision-making process. The benefit of using SW technologies in such systems is to integrate heterogeneous formats of knowledge sources together. It also helps to handle complex and large datasets in order to share and reuse knowledge. Although the system is not scalable enough in a large clinical setting, it provides a flexible architecture.

Bio-DASH [24] is a SW-based prototype of a drug development dashboard. In this type of KB-CDSS, users use an RDF model to diagnose diseases, compounds, drug progression stages, molecular biology, and pathway knowledge. This system addressed the problem of sharing heterogeneous knowledge in the KB-CDSSs. To tackle this issue, the authors proposed a SW-based framework using RDF or OWL languages to describe objects and the relations between them. The framework supports data integration and user authorization. The proposed method suffers from the lack of having an appropriate platform for sharing and aggregating knowledge. High memory usage is another drawback of the proposed model.

A number of papers described a proposed clinical practitioner guideline (CPG) KB-CDSSs [1,17,25,26]. The main idea behind these series of papers is to integrate different types of ontologies such as the domain ontology, CPG ontology, and patient ontology by developing a knowledge-centric system. This system, which has been developed for the community of Breast Cancer Follow-up (BCF), contains three main components including (1) paper-based BCF CPG computerization, (2) ontology development, and (3) executing BCF CPG in a logic-based engine. Technically, this structure helps to reduce the workload of the specialist cancer center. The simple and flexible usage of data publishing and integration along with user interaction are the advantages of using SW technology in these frameworks. However, the proposed system is quite generic and needs to be validated in different situations.

[27] offered an ontology-based approach for predicting the risk of hypertension and diabetes in KB-CDSSs. To this aim, the authors used ontologies for representing patient medical profile and improving an inference mechanism for clinical decision making.

[28] proposed a SW-based KB for clinical pharmacogenetics in order to manage data. The KB has been developed by utilizing SW standardizations such as RDF and OWL. The OWL ontology contains the details of drug product labels of pharmacogenomics information. The advantages of using SW technologies have been highlighted in this study. The ontological-based structure can increase the likelihood of successful long-term maintenance and growth of KB. They are also beneficial for handling an enormous amount of datasets and share and reuse ontological concepts.

The Cleveland clinic supported a project called Semantic-DB [29] that proposed a framework to collect, store, and reuse knowledge to support sufficiency, flexibility, and extensibility of different clinical data. The reliability of research results and the accuracy of quality metrics are the addressed issues in this paper. The proposed model contains three main components: (1) content repository, (2) query interface, and (3) data production. The results obtained by the method show that the system can guarantee the quality of care measurements. It has also reduced the duplicate efforts as well as imposing transparency to deduct errors in the reported data. For the future, this research needs to be improved from different aspects such as ontology alignment, maintaining semantic alignment, and improving performance.

Finally, [30] focused on answering the question of “how the SW tools such as ontologies and rules can be applied to connect the medical and oral health (M-OH) domains by developing a KB.” The KB can be reused by the medical information systems for semantic interoperability and reasoning process. The system has been developed by utilizing OWL and SWRL rules. According to the results, effectiveness in reasoning, comprehensive cross-domain KB, and cross-domain communication are the strengths of the proposed system.

#### Semantic Web (SW) Services

COCOON glue [21] is an SW-based service to integrate complex eHealth services. It uses Web Service Modeling Ontology (WSMO) with an open source f-logic inference engine called Flora2 to run over an open source deductive database system. This system aims at reducing medical errors and developing an efficient Web service management system to publish, discover, and compose services. This system has two main advantages: (1) providing a clear separation between the ontologies and (2) preparing good performance. The major weakness of this study is related to the use of f-logic technique for defining similarity metrics. The f-logic is a set of predefined rules for making deductions. Basically, methods developed by f-logic technique are not scalable enough and cannot be applied on the large volume of data.

ARTEMIS [31,32] is a project supported by the European Commission based on the SW services using the OWL. The structure of this system is similar to COCOON. It aims to describe the semantics of Web service functionality. It also supports the semantic meaning of messages or documents exchanged through Web services. As previously mentioned, using SW technologies not only enables health care services to easily interact with each other but also helps to integrate data across the clinical Web service by using semantic annotations. However, this system does not provide a secure platform for protecting data.

[15] addressed the interoperability problem in both the domain of data integration and heterogeneous systems. They proposed a SW-based service framework to tackle the problem and empower the semantic interoperability among health care systems.

Despite improving health care quality, sharing and extracting knowledge in a heterogeneous environment is the most popular limitation among KB-CDSSs. Therefore, [33] proposed a sharable KB-CDSS that meets this challenge. This system has a SW service framework to identify, access, and leverage independent and reusable knowledge modules located in the central KB. The knowledge modules are defined by the ontological model, terminologies, and representation formalisms to support sharable KB-CDSS. Their contributions consist of representing unified knowledge and patient data in heterogeneous domains, knowledge integration and data interoperation, and semantic development of sharable knowledge for automated knowledge acquisition. This system has been evaluated by two applications including model-level and application-level evaluation. The coherent knowledge representation is confirmed by model-level evaluation. The high accuracy and completeness is validated by application-level evaluation. These evaluations show this system is feasible and useful in providing sharable and reusable knowledge for the purpose of diagnosis in decision making. It is also offers time-saving benefits and cost effectiveness in comparison with the other KB-CDSSs. The system improves the maintainability and scalability of systems to contribute with the other KB-CDSS.

[34] suggested a SW-based framework to support reasoning to remedy diagnostic errors. The authors believe that diagnostic errors are derived from flawed reasoning, incomplete knowledge, faulty information discovery, and inappropriate decision making. This approach contains a case-based fuzzy cognitive mapping to support diagnosis. The framework also evaluates the clinical knowledge for decision making by using Bayesian belief networks. The reasoning methods for this framework used statistical approach to solve the diagnosis issues and enhance the efficiency of the system. The reasoning methods used in this approach are implemented by using the SW tools such as Notation 3 or RDF and Euler Sharp inference engine. The strength of this system is in handling approximate reasoning, incomplete information, control rules for clinical conditions, and patient profiling. This approach is in the first stages of development for implementation. It needs to be tested with larger datasets and allowing updating of the system by integrating new knowledge.

Another study proposed by [35] developed a multiagent framework called MAPP4MD to provide a privacy preserving mechanism for clinical data in a heterogeneous environment. In this study, each agent utilizes ontologies and SW technologies to apply reasoning for a privacy-preserving algorithm. This approach supports data integration and sharing among agents in the various environments for knowledge discovery. The evaluations of this system show that the distributed multiagent framework is flexible. One of the benefits of this approach is to improve data sharing for medical research, population-level analysis, and evaluation of population-level in health care activities. Although this framework works fine in the limited datasets, it needs to be checked in the larger datasets to show its scalability.

[36] addressed the problem of standalone KB-CDSS and having a universal KB-CDSS. The authors developed a semiautomated approach to discover, select, and compose KB-CDSSs available as Web services. The proposed system is at the elementary level and needs to be implemented and validated. The lack of identifying formalized semantics attached to the services is an obvious challenge for this research.

### Lack of Semantic Analysis

The reviewed papers in this section have proposed two SW frameworks to improve the lack of semantic analysis. They consist of knowledge engineering technique and logic reasoning structure. The main goal of these papers is to improve knowledge acquisition process in the KB-CDSSs by utilizing SW technologies.

#### Knowledge Engineering Technique

Many of the non–SW-based KB-CDSSs suffer from the lack of automatic analysis systems. This issue can be addressed by using SW technologies. A knowledge engineering approach was taken in [37,38] for detecting Alzheimer’s disease to help physicians to detect it in the early stages by using multidisciplinary knowledge and reasoning over the underlying KBs. In this paper, researchers used ontologies (eg, MIND ontology, Semantic Web applications in neuromedicine [SWAN] ontology, and systematized nomenclature of medicine-clinical terms [SNOMED-CT]). Although this project needs to be tested on the larger ontological domains, the authors improved the accuracy of results for further decision-making processes. In 2012, the system improved for discovering new knowledge and generating new rules for clinical decision making [39,40]. It is important to mention that physicians take advantage of this system to help patients discover relevant knowledge for Alzheimer’s disease diagnosis. This KB-CDSS not only works in the Alzheimer’s disease domain but also supports the other domains such as cancer.

The authors in 2013 [40] proposed a more generic software architecture called S-KB-CDSS to solve some of the challenges of KB-CDSSs. They improved the system by adding new tasks to the system such as diagnosis, prognosis, treatment, evolution, and prevention. It helps the system to integrate and reutilize clinical workflow of KB-CDSSs. They mentioned that discovering new knowledge methods in a previous study was implicit and that they want to solve other challenges of KB-CDSSs. They mentioned that because of the nature of a system that is based on knowledge model provided by a team of domain experts, classical validation is not possible in this stage. Therefore, they assumed that the system is correct. They validated their system by comparing system decisions with end-user decisions.

In another paper, [41] developed a model for semantic enhancement of KB-CDSS by using knowledge engineering technique to express the domain of knowledge and the patient data in a unified model. The architectures included four different phases: (1) knowledge acquisition, (2) knowledge representation, (3) knowledge application, and (4) knowledge evaluation. The main motivations for developing such architectures were to handle multidisciplinary and heterogeneous platforms. The authors claimed that their system was useful because it could reduce the reduplication of data in the KB. However, it needed to support experience based reasoning, as well as bridge the gap between semantic health care KB and existing knowledge representation model.

Another knowledge engineering approach that aims to improve the performance of KB-CDSS was proposed by [42]. This approach answers queries by integrating deterministic and plausible knowledge from heterogeneous environments. Researchers in this study used SW technologies to leverage reasoning and extend the coverage of a medical KB. Extending the coverage of medical KB, by considering potential correlations between decisional attributes is useful, especially when KB-CDSSs need to have complete knowledge for decision making.

There is some rationale for using SW technologies in this approach, such as data management, description logic (DL)–based inferring methods, and the opportunity to support plausible reasoning. Moreover, using ontology inference and conceptual similarity check, improves the accuracy of reasoning in the system. The result of the system evaluation shows that this multi strategy approach improves knowledge coverage of clinical KB and helps to have better diagnostic process for complex diseases. In addition, inferred knowledge can be used in future decision making.

#### Logic Reasoning Structure

[43] proposed a knowledge-based preoperative decision support system to assist health professionals in secondary care in the preoperative assessment of patient before elective surgery. In this system, the authors applied SW technologies such as OWL and logic reasoning to develop an automatic analysis system. The system attaches patient information to the medical context. However, the collected information from patients is still a kind of “coarse-grained” information and needs to be transformed into a “fine-grained” model.

[44] proposed a personalized treatment flow without user intervention. The method was developed by using fuzzy decision tree, fuzzy rules, and SWRL. The advantages of such systems are to provide a user-friendly environment to improve memory performance and to reduce the time on patient care. The scalability of the proposed model is still under investigation.

SeDelo [45] suggested a computer-aided diagnostic system to help experts and nonexperts to recommend the clinical diagnosis. In this study, the authors developed a KB-CDSS by utilizing SW technologies and description logics to diagnose diseases by using symptoms, signs, and laboratory tests. This system is more efficient and accurate in decision-making processes compared with previous systems proposed by the same authors. Although this method achieves a better result in terms of the accuracy of the system, it is still not scalable enough and needs to be developed for the rule description process.

## Discussion

### Principal Findings

SW technology and its applications are useful since in principle they can deal with data from multiple sources and facilitate machine-machine communication. The SW is an effort to make knowledge on the Web both human-understandable and machine-readable. There is no need to provide a database schema for sharing data since it has its own universal data structure and can be used among knowledge sources. SW technologies and their features such as semantic interoperability, knowledge integration, and knowledge reusing to upgrade and transform old applications into modern and intelligent models [46].

In order to make well-informed decisions, health care applications need to be able to discover knowledge among many heterogeneous KBs. Having diverse data models and formats lead researchers to use SW technologies to facilitate data integration processes. SW technologies allow researchers to analyze incompatible biological descriptions in one unified format. For example, using SW technologies helps to mesh datasets about protein-protein interaction to reveal obscure correlations that could help identify promising medications [7].

In the context of KB-CDSSs, different issues have been improved by utilizing SW technologies such knowledge acquisition and data collection, and data integration of clinical systems. In this paper, we have reviewed and highlighted the issues of knowledge acquisition improved by SW technologies. The review shows some of the potential approaches of SW technology in supporting KB-CDSSs.

### A Proposed Model for Semantic Web (SW) Use in Knowledge-Based Clinical Decision Support System (KB-CDSS)

**Figure 3:**
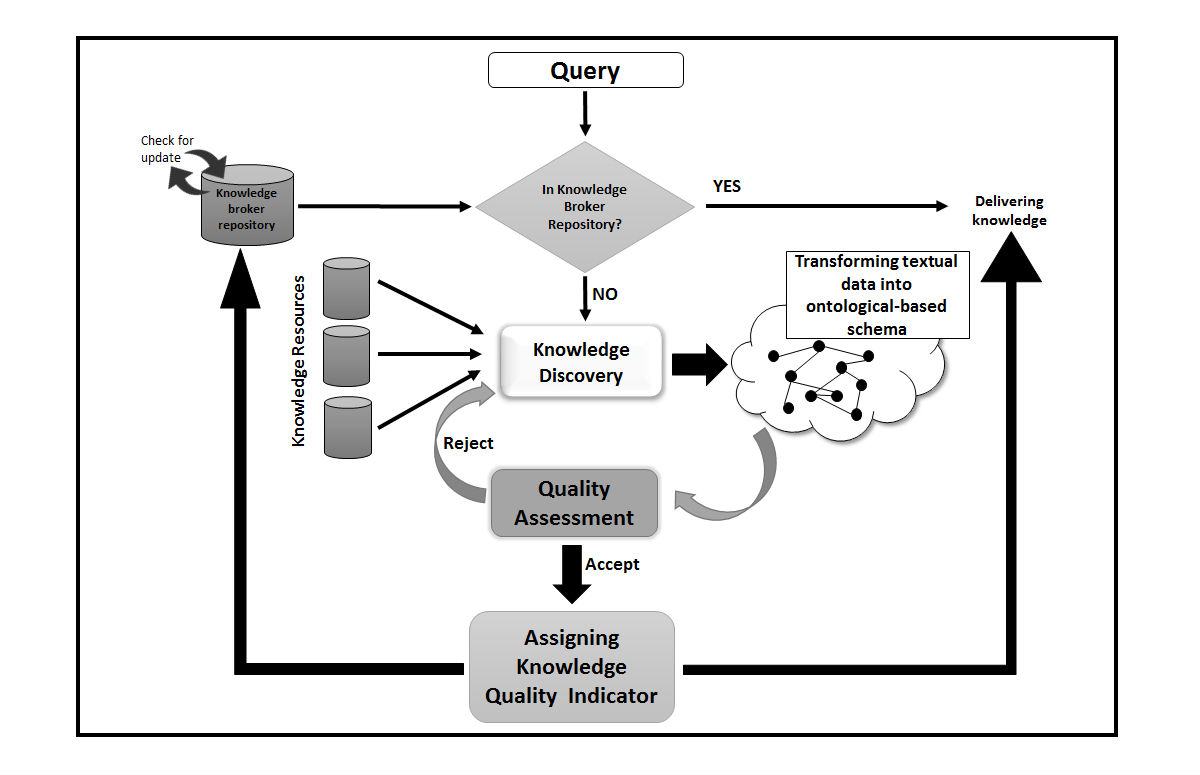
A proposed model for using Semantic Web (SW) in knowledge acquisition for knowledge-based clinical decision support system (KB-CDSS).

To discuss how the knowledge can be discovered and updated with the SW technologies, we have proposed a knowledge broker framework to apply in a KB-CDSS [47]. In this framework, we focus on assessing clinical knowledge and delivering high-quality knowledge for a KB-CDSS. The overall framework is shown in Figure 3. The proposed model contains five major parts.

#### Knowledge Discovery

After receiving a query, the knowledge broker will check the existing knowledge in its repository to find a related result. If the knowledge exists in the repository, the system will deliver the knowledge immediately, if it does not exist, the new knowledge will be extracted from electronic knowledge resources (especially PubMed) based on query characteristics.

Note that in the system, there is a knowledge repository, which records all of the extracted knowledge with a knowledge quality indicator. The knowledge quality indicator can support relevancy, currency, and accuracy of knowledge to use in decision making. The knowledge repository will check the quality of knowledge regularly to provide high quality knowledge every time. If the knowledge needs updating, the knowledge broker improves the knowledge and sends it back to the repository.

We assume that all of the knowledge that we have used is of the OWL or Semantic format.

#### Constructing Knowledge

In this step, the extracted knowledge will be converted to the ontology format and annotated by other information to enrich the knowledge. To achieve this, Protégé Ontology editor has been utilized. In the knowledge discovery step, the knowledge broker may extract several items of knowledge that are useful. Therefore, we may use their information to annotate the extracted knowledge. The output will be an enriched knowledge for the system.

#### Quality Assessment

This step is related to checking the quality of the knowledge to ensure it is useful for decision making. We may use different metrics to check the quality of knowledge.

#### Assigning Knowledge Quality Indicator (KQI)

In this step, a knowledge quality indicator will be assigned to the knowledge item to show how much knowledge is qualified. The knowledge quality indicator (KQI) can support the approval for knowledge quality. It will be more useful to have a knowledge quality indicator when reusing the knowledge in the future.

#### Updating Knowledge Repository and Delivering Knowledge

Finally, the high quality knowledge will be sent to the knowledge repository to be used again. It will deliver to the KB-CDSS for decision making respectively.

### Limitations and Future Directions

Although SW technologies improve the problem of knowledge acquisition in KB-CDSSs, there are still some issues that have not been considered yet. For example, in the context of KB-CDSSs, most existing methods do not properly evaluate the quality of extracted knowledge. Here the question is how to make sure that the knowledge used by KB-CDSSs is reliable.

Conventional search engines cannot evaluate whether the knowledge is accurate, reliable, and relevant in the case of comorbidities. Inappropriate knowledge can have negative effects on the decision-making process. Therefore, there is a need to propose new methods to check the quality of extracted knowledge using SW technologies for KB-CDSSs.

There are also some limitations in applying SW technologies to systems such as KB-CDSSs. Apart from using and managing personal data and knowledge, the privacy issues around using SW could be a significant problem in such systems, primarly because everything that is published online will be shared using SW technologies. Another issue that can be problematic for applying SW technologies can be resouce requirements to support complete features of SW. The SW technology may need some specific resources to work; however, some of them may not exist in the current environment. There is still a very long way to go before the SW dream becomes true and changes the information society and the information economy. SW technologies aim to convert syntactical structure to semantical structure. They also aim to facilitate the process of retrieving information to delegating tasks. In this regard, health informatic experts need to make efforts to utilize SW technologies in the body of CDSSs.

In addition, we have identified a number of still-open questions: (1) Are KB-CDSS approaches more effective than machine learning systems, or should they be combined with them? (2) How can the quality of knowledge discovered by a SW approach be evaluated? (3) Is it possible or even desirable to use an SW approach to automatically update KB-CDSSs? and (4) Should knowledge sources conform to a particular standard in order to support SW-based knowledge acquisition, and would this justify the overhead associated with such work?

### Conclusions

The rise of precision medicine is also a key driver in the need to identify both knowledge and data from heterogeneous sources [48,49]. The aim of this systematic review paper is to highlight the importance of using SW technologies for improving the knowledge acquisition issues in the context of KB-CDSSs. In this paper, the potential for using SW technology has been described. We have categorized the recent knowledge acquisition issues of KB-CDSSs improved by SW technologies into 2 main groups including format and data heterogeneity and lack of semantic analysis. In this regard, we have reviewed the recent related work in this context to highlight the necessity of using SW technologies in the body of current KB-CDSSs.

As discussed previously, the existing health care search engines (ie, PubMed and Clinical Trials) do not comprehensively extract and identify high quality knowledge for serving in the KB-CDSSs. The ever-growing amount of clinical knowledge makes the process of extracting high quality knowledge increasingly difficult. None of the reviewed papers have addressed the issue of quality assessment for KB-CDSSs. For future work, we aim to develop an automatic system to measure, extract, and rate the high quality knowledge for KB-CDSSs [47]. Such a system should be able to support knowledge brokers to extract and rate knowledge from multiple heterogeneous sources (ie, PubMed and other sources) to keep KB-CDSSs current and provide optimal decision making. There is also the possibility of integrating such systems with a precision medicine–based approach [49] to allow a KB-CDSS to discover appropriate cases and outcomes that may need to be included in rule revision.

## Abbreviations

AD: Alzheimer’s disease
BCF: Breast Cancer Follow-up
CDSS: clinical decision support system
CPG: clinical practitioner guideline
KB: knowledge base
KB-CDSS: knowledge-based clinical decision support system
KQI: knowledge quality indicator
M-OH: medical and oral health
OWL: Web Ontology Language
SNOMED-CT: systematized nomenclature of medicine-clinical terms
SW: Semantic Web
SWRL: Semantic Web Rule Language
